# Climate and nature emergency: From scientists’ warnings to sufficient action

**DOI:** 10.1177/09636625221100076

**Published:** 2022-05-30

**Authors:** Alan Cottey

**Affiliations:** University of East Anglia, UK

**Keywords:** climate emergency, ecological crisis, ecological emergency, nature emergency, public understanding of science, scientists warning

## Abstract

Scientists’ warnings of a climate and ecological emergency have been published recently. They have been criticised as being unattractive to non-scientists. Here, the criticisms are reviewed and comments presented. The path is long between primary research and the daily concerns of hard-to-reach people (e.g., those who are impoverished). It is enough that expert scientists express their findings accurately and intelligibly to all who are receptive. Outside the ranks of the specialist experts, there are many – intellectuals of all kinds, journalists, politicians, business people, and concerned citizens – who are well placed to contribute to the generation of a worldwide groundswell of practical action. The full range of discourse on the ecological issues is divided into four registers: used in primary research; dissemination of specialists’ thinking to non-specialists; discussion with those engaged in public affairs; and discussion with those who face obstacles to becoming engaged with the issues.

It is widely acknowledged that humanity faces a climate and ecological emergency, and that our responses to date are far too slow and cautious. The mismatch between danger and appropriate action is partly a problem in the communication of science, to which *Public Understanding of Science* scholars have made numerous research contributions. This brief essay, from outside the *PUS* and similar research communities, is offered as a contribution to an urgent practical problem. It considers a very specific class of publication.

## 1. The scientists’ warnings

2020 saw noteworthy growth in the number of *scientists’ warnings* – admonitions, often directed explicitly to humanity, about the dire state of our planet’s ecology and the role of human activity in this. What may be considered a distinctive genre has its origin in a leaflet produced by the Union of Concerned Scientists (UCS). *World Scientists’ Warning to Humanity* (Union of Concerned Scientists, 1992) opens with the blunt warning ‘Human beings and the natural world are on a collision course’. It asserts that the environment is suffering critical stress and that we must accept limits to consumption and population.^1^ At that time, there was little chance of so overtly value-laden a message being accepted in the source-referenced and peer-reviewed scientific literature. Being grey literature, it was in the course of time largely forgotten.^2^ But the issue had not gone away.

## 2. A second notice

In 2017, 25 years after its original publication, the UCS warning was brought to renewed attention with the article *World Scientists’ Warning to Humanity: A Second Notice* (Ripple et al., 2017). In the interval, knowledge about global ecology, including human influences, had become firmer – and even more disturbing. It was possible for an overview, with quantitative data, to be published in a highly respected scientific journal. The core of the article comprises graphs showing the course, from 1960 to around 2015, of nine indicators of global ecological stress. All of these indicators except one, on ozone depleters, show marked increase of stress. In nearly every case, the adverse trend is roughly the same after 1992 as before. Thus, during a quarter-century opportunity, humanity did not heed the UCS warning.

Since those first two warnings, there has been an increasing flow of scientists’ warnings on facets of the general problem. An early and notable one of these is *World Scientists’ Warning of a Climate Emergency* (Ripple et al., 2020), with a form similar to that of the *Second Notice*. It displays trends over time in the intensity of 15 human activities with global climate impact, and 14 climate trends. Although the general direction of these trends is adverse, the authors in their conclusion do also point to a recent surge of concern on the part of governmental bodies, schoolchildren, litigants and citizens. Other alarming ecological imbalances have also been the subject of scientists’ warnings, especially from 2020. These include freshwater biodiversity (Albert et al., 2021), microorganisms (Cavicchioli et al., 2019) and many more.^3^ The scientists’ warnings that are the subject of this essay differ markedly from earlier warnings stretching back through history, of which the Russell–Einstein manifesto (Russell and Einstein, 1955) and *Silent Spring* (Carson, 1962) are notable examples.

A major recent development of the genre is notable as a foray from the natural sciences into the highly politicised territory of affluence and survival. *Scientists’ Warning on Affluence* (Wiedmann et al., 2020) makes strong claims – that by far the largest factor determining ecological impact is consumption and that consumption is more aptly labelled affluence. Time will judge whether the weight of natural science has successfully been brought to bear on a subject (affluence) that is generally studied in a sociological and economic context.

## 3. To sensitise humanity

The scientists’ warnings go beyond accounts, intelligible to a wide readership, of the current scientific consensus on reliable knowledge about global ecological overload. They attempt to sensitise humanity as a whole to the implications of this knowledge and they enter the domain of policy, recommending radical changes of behaviour in all aspects of human activity. By 2017, the time of the *Second Notice*, it was clear that humanity was not addressing the issues of sustainable living in the way that the 1992 UCS warning deemed necessary. The warnings were evidently unwelcome. To some extent, people deployed psychological defences (inattention, distraction, data selection, denial) against disagreeable news. Perhaps more important, the news was known to be likely true, but psychic numbing (Slovic, 2020) and inertia inhibited appropriate action. Individuals and groups could respond to clear and present danger, but not to warnings of dangers that were global, novel and (largely) about a relatively remote future.

## 4. Scholarly responses

The three principal scientists’ warnings – *UCS* (Union of Concerned Scientists 1992), *Second Notice* (Ripple et al., 2017) and *Climate Emergency* (Ripple et al., 2020) – have attracted responses from natural scientists, social scientists and humanities scholars. Some of these responses are endorsements (Skubała 2018a, 2018b) and the others are sympathetic to the aims but critical of the warners’ methods, implicit assumptions, language and effectiveness (Bull et al., 2017; Hartman and Oppermann, 2020a; Kayal et al., 2019). There is objection to the tone of the *Second Notice*, which is claimed to have been conducted in a top-down manner, and to be saying virtually nothing about poor people (Bull et al., 2017). The *Second Notice* is also accused (Kayal et al., 2019) of addressing symptoms rather than root causes, normalising the Western lifestyle and neglecting prevailing inequalities.

The inaugural issue of *Ecocene* (Hartman and Oppermann, 2020a) has the theme *Environmental Humanists Respond to the World Scientists’ Warning to Humanity.* The editors’ introduction includes an overview (Hartman and Oppermann, 2020b). Two reservations run throughout the collection. One is expressed by Slovic (2020: 46) – ‘I find that the “World Scientists’ Warning” delivers a flood of convincing information about the direness of our climate predicament but does so in a way that smothers . . . the salience of the warning itself’. The other prominent reservation (Hartman and Oppermann, 2020b: 10–11) is that ‘people are much more likely to be influenced and motivated by storytelling, music, and art than by scientifically rigorous presentations of data’.

Specific criticisms include: humanity is not a unified entity (Braidotti, 2020; Yearley, 2020: 19–21); people are vulnerable to psychic numbing when inundated with abstract information (Slovic, 2020: 43); cautionary assertions may be perceived as finger-wagging (Slovic, 2020: 50); and globalist arguments are not easy to reconcile with life as it is lived (Foote, 2020: 55). Other offerings include: climate change should no longer be seen as a purely global problem but must enter the heart of local economies (Blanc, 2020: 134); where geoscientists speak of a planet in crisis, humanists speak for a world where the societal responses to crisis need to be narrated and communicated widely (Castree, 2020: 37); lessons can be learned about the poignant use of language (Slovic, 2020: 45); and those in the environmental humanities, as well as natural scientists, are called to play ‘the watchman’s part’ (Szerszynski, 2020: 97–98).

## 5. Comments on the scholarly responses

The criticisms (Bull et al., 2017; Hartman and Oppermann, 2020a; Kayal et al., 2019) fully respect the warners’ knowledge but are often severe on their communication skills. The warnings can, however, be afforded a more sympathetic reading. The efforts of Ripple et al. (2017) and Ripple et al. (2020) may not have turned human culture away from a collision course, but they have raised awareness of the UCS warning and its manifold aspects (climate, oceans, forests, biodiversity and more). This is attested by many responses in the scientific and scholarly literature.^4^

In terms of technical accuracy, the scientists’ warnings have a good track record. In no particular have any of the warnings been shown to be ‘false alarms.’ Even in terms of being heard, the warnings have been quite successful, the inadequacy of business-as-usual now being widely exposed (Kramer et al., 2019). The real failure is that, except in the one case of stratospheric ozone depletion, *collective actions to avert danger have been far short of what is needed.* As long as economic growth remains a fetish, identified with human well-being, the global ecological overload will become worse. There are, however, recent signs of cracks in neoliberal hegemony (Kramer et al., 2019).

Some critics of the scientists’ warnings doubt whether telling the scientific truth about climate change and the global ecological crisis is really enough to lead people to action (Armiero, 2020: 150). But the scientists cannot, more than others with agency, be held responsible for the lack of an adequate global political reaction. Might the warning scientists, in their anguish, have taken on too much by directly addressing all humanity? From that point of view, the role of the world’s most informed specialists is to present accurate simplified accounts of what they know to a wider constituency comprising those who have some interest in and knowledge of science.^5^ The many graphs in *Second Notice* and *Climate Emergency* can be considered in just this way – as a bridge between the specialists’ knowledge base and that wider constituency. The important task of reaching further, to those who have no appreciation of science, but whose lives are affected by it, lies outside the skill-set of most scientists. That task is principally for scholars, journalists and other writers. In this analysis, the responsibility for warning humanity at large is spread widely and is bearable.

## 6. Sufficient action

**Figure 1.**
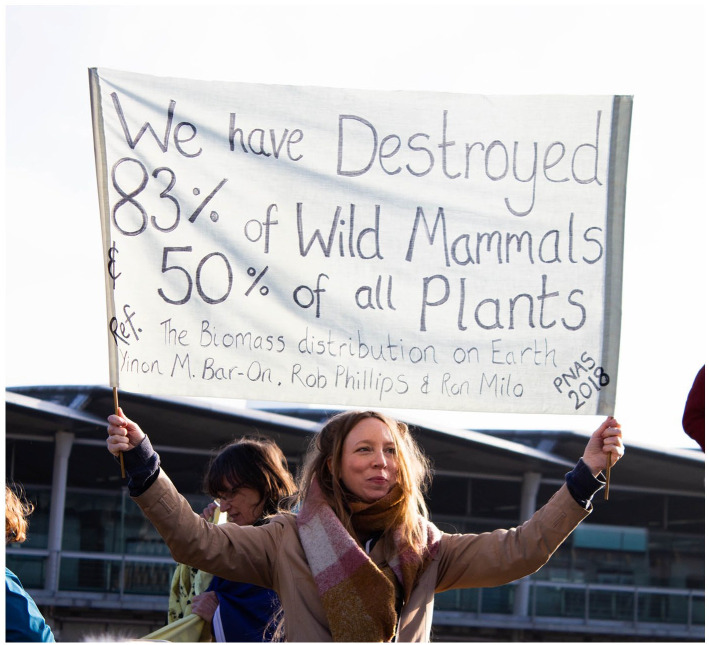
Peer-reviewed primary research papers are not infrequently cited by activist demonstrators, especially when concerned with climate change or biodiversity loss (Hawkins, 2018).

The *Second Notice* showed how insufficient were humanity’s responses to the global ecological emergency. The more recent *Climate Emergency* warning shows the insufficiency continuing, although it does also see some recent positive developments from governmental bodies, schoolchildren, litigants and citizens. Other positive signs are that scientific conclusions about climate change and biodiversity loss are steadily becoming more reliable and accurate (and thus more persuasive); and that, in the current pandemic conditions, science has gained enhanced respect (Figure 1).

Expert scientists’ warnings will no doubt continue for some time, on further subjects and with improved data. But the greatest problem for now and the foreseeable future is *appropriate and sufficient action*. Asking the warning scientists to improve their communication skills, to the point where their testimony becomes irresistible, is too much. It is enough that those scientists refine and promote the task that only they can do – producing reliable, relevant knowledge and starting the dissemination process with warnings written for receptive non-specialists. Thus, the first stage is to inform and energise a large cadre of scientifically literate people about the extreme scale and urgency of the danger. The second stage requires the creation of a worldwide groundswell – the active participation of an even larger class of concerned citizens. The input of the few specialist scientists who also have outstanding communication skills is of course welcome. But the huge challenge requires also the skills and energy of all others with something to offer – intellectuals of all kinds, journalists, politicians, business people and concerned citizens. No single linguistic register is adequate for the totality of discourse needed for addressing the challenge.

As a response to the scholars’ criticisms of the scientists’ warnings, it is here proposed that discourse on the great range of the ecological issues can usefully be divided along a line, albeit with fuzzy boundaries, into four registers. The first of these is used in the practice of primary scientific and scholarly research, and is specialised and technical. The next register is used in the dissemination of specialists’ thinking to non-specialists beyond their circle and is at the level of scientifically literate non-specialists. The third register is used for discussion with those explicitly engaged in public affairs and is the medium of the interplay between scientific truth claims and social values. The fourth register is for discussion with a hard-to-reach group – those who face obstacles to becoming explicitly engaged in the ecological issues. These obstacles may be short-term, such as lack of necessities, or medium-term, such as chronic overwork, or long-term, such as consumerist acculturation. Removing these obstacles presents the most difficult challenge in the entire project of developing a global culture of sufficient action.

An easier but still important challenge in the fourth stage is the further development of everyday and empathic language for dialogue with those who are currently hard to reach. A development in the fourth register has occurred recently with the increasing use of the phrase (IPPR Environmental Justice Commission, 2020) *climate and nature emergency* (or *crisis*). The novelty here is the replacement of the formal terms *ecological* or *biodiversity*, usual in the other registers, by the everyday term *nature*. The more precise formal terms are suitable in their place, but replacing them in the fourth register by *nature* may turn out to be a significant contribution to popular outreach. *Nature emergency* has been taken up with energy by such green campaigners as Climate Scotland (2021).

## 7. Ultimately, all can contribute

This essay takes on board the points made by the critics of the Scientists’ Warnings. It proposes a flat-line structure within which those with agency – ultimately all – can contribute to sufficient responses to the climate and nature emergency. First must come a groundswell for us humans taking a modest, just and sustainable place within life on earth. Then, those who currently are not in a position to do so may also be empowered to think and act with global and long-term reach.

## Notes

1. On the origin of the *World Scientists’ Warning to Humanity*: In February 1990, the UCS released to the press its *Appeal by American Scientists to Prevent Global Warming*, the signatories including over 700 members of the National Academy of Sciences. The Appeal is reprinted in Kendall (2000: 183–188). In February 1992, the Royal Society of London and the US National Academy of Sciences, in the run-up to the forthcoming (June) Rio Earth Summit, issued a joint statement on *Population Growth, Resource Consumption, and a Sustainable World*. At the end, the statement declares ‘the future of our planet is in the balance’. Dieoff.com provides a transcription of this not-easy-to-find statement (Atiyah and Press, 1992). Also early in 1992, the UCS had a leading role in conveying to the UN Intergovernmental Committee the thoughts of environmental NGOs on the objectives and priorities of the Earth Summit (Kendall 2000: 180, 189–191). Freestone (1994) wrote of the Earth Summit ‘Assessments of Rio were mixed; some hailed it as an overwhelming success, some as an overwhelming failure’. Achievements, by way of global agreement, fell far short of what the NGOs had proposed.

2. But not entirely forgotten. Pre-2017 references to the UCS warning include Hallman 1995; Sugunasiri 1997; Ehrlich and Ehrlich 2009, 69; Rapport et al. 2009, 3; White et al. 2011, 472; Skubała 2016, 32.

3. Including wildfire (Coogan et al., 2019), wetlands management (Finlayson et al., 2019), subterranean ecosystems (Mammola et al., 2019), medicinal plants (Applequist et al., 2020), insect extinctions (Cardoso et al., 2020), ethology (Goymann and Küblbeck, 2020), food webs (Heleno et al., 2020), large lakes (Jenny et al., 2020), the Mediterranean (Kopnina et al., 2020), human population (Lidicker, 2020), invasive alien species (Pysek et al., 2020), avoiding a ghastly future (Bradshaw et al., 2021), conservation physiology (Madliger et al., 2021) and wildfire and water supply (Robinne et al., 2021).

4. Responses additional to those discussed here are on – the most important message (Skubała, 2016), warnings without power (Dror, 2018), economic growth (Pacheco et al., 2018), a conservation economy (Ripple et al., 2018), protection of our planet (Skubała, 2018), the scientist as creator and destroyer (Cuhra, 2019), scientists must act on own warnings (Gardner and Wordley, 2019), pathways for scientists (McDonald et al., 2019) and population (Washington et al., 2020).

5. A review of research on accuracy, with emphasis on media representations of science, is given in Hansen (2016).
